# Quantum Transport and Nano Angle-resolved Photoemission Spectroscopy on the Topological Surface States of Single Sb_2_Te_3_ Nanowires

**DOI:** 10.1038/srep29493

**Published:** 2016-09-01

**Authors:** Yulieth C. Arango, Liubing Huang, Chaoyu Chen, Jose Avila, Maria C. Asensio, Detlev Grützmacher, Hans Lüth, Jia Grace Lu, Thomas Schäpers

**Affiliations:** 1 Peter Grünberg Institute (PGI-9) and JARA Jülich-Aachen Research Alliance, Research Centre Jülich GmbH, 52425 Jülich, Germany; 2 Department of Physics and Astronomy and Department of Electrophysics, University of Southern California, CA 90089, Los Angeles, USA; 3 Synchrotron SOLEIL, L’Orme des Merisiers, Saint Aubin-BP 48, Gif sur Yvette 91192, France

## Abstract

We report on low-temperature transport and electronic band structure of *p*-type Sb_2_Te_3_ nanowires, grown by chemical vapor deposition. Magnetoresistance measurements unravel quantum interference phenomena, which depend on the cross-sectional dimensions of the nanowires. The observation of periodic Aharonov-Bohm-type oscillations is attributed to transport in topologically protected surface states in the Sb_2_Te_3_ nanowires. The study of universal conductance fluctuations demonstrates coherent transport along the Aharonov-Bohm paths encircling the rectangular cross-section of the nanowires. We use nanoscale angle-resolved photoemission spectroscopy on single nanowires (nano-ARPES) to provide direct experimental evidence on the nontrivial topological character of those surface states. The compiled study of the bandstructure and the magnetotransport response unambiguosly points out the presence of topologically protected surface states in the nanowires and their substantial contribution to the quantum transport effects, as well as the hole doping and Fermi velocity among other key issues. The results are consistent with the theoretical description of quantum transport in intrinsically doped quasi-one-dimensional topological insulator nanowires.

Three-dimensional topological insulators (TI) such as Bi_2_Te_3_, Bi_2_Se_3_, or Sb_2_Te_3_ 1,2,3 constitute a new class of quantum materials with topologically protected metal-like surface states. For bulk crystals, a significant drawback arises from the presence of high carrier concentration due to intrinsic defects in the bulk, which mask the characteristic non-trivial topological surface transport. Mesoscopic structures such as ultra-thin films and nanowires (nanoribbons) have been envisaged as promising platforms to explore intrinsic surface states, whose decoupling from bulk states is enhanced due to the high surface-to-bulk ratio and the effective tuning of the Fermi level. Theoretical and experimental reports also point out the novel physics in TI nanowires, as well as at their interfacing with magnetic materials or superconductors, e.g. proximity effect, spin manipulation, and Majorana fermions^4^,^5^,^6^. However, on TI nanowires many significant experimental challenges still remain, such as the intrinsic defects (disorder), control of the stoichiometry and understanding of the electronic band structure. Particularly in transport, TI nanowires offer an outstanding playground to prove and exploit TI surface states via the Aharonov-Bohm (AB) effect^7^,^8^,^9^,^10^,^11^,^12^,^13^,^14^,^15^. When a magnetic field is applied along the main axis of the nanowire (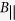) [cf. Fig. 1], the wavefunction of metal-like surface states around the cross-sectional area of the nanowire (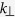), acquires a phase-shift of 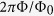, where Φ_0_ = *h*/*e* ≈ 4.14 * 10^−15^ T m^2^ is the magnetic flux-quantum and Φ defines the magnetic flux. In TI nanowires, Dirac fermions completing the loop around the nanowire (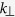) are subject to an additional *π* phase accumulation (Berry phase) due to the helical spin-momentum coupling. This implies that at zero or any integer multiple of the flux quantum Φ_0_ the antisymmetric wavefunctions cancel each other leading to the opening of an energy-gap at the lowest subband of the surface state energy spectrum. For any odd half-integer multiple of Φ_0_ there is a total phase-shift of 2*π*. Hence, the wavefunction restores its original symmetry forming the gapless Dirac linear dispersion in the energy spectrum, as shown in Fig. 1. For all other values of Φ the breaking of time-reversal symmetry prevents the TI surface states. Consequently, an increase of the applied magnetic field 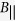, gives rise to resistance oscillations with a characteristic period of Φ_0_ originating from the TI surface states^16^. However, the observation of AB-like oscillations is by itself no proof for transport in the topological surface states, since they have also been observed for semiconducting nanowires with a surface accumulation layer due to band bending^17^. In fact, the possible impact of band bending on transport at surfaces of topological insulators is one issue which has been discussed in literature as well^18^,^19^,^20^. The lack of supportive clear and direct manifestations of those states in TI nanowires based on the analisys of their electronic band structure, limits the discussion on quantum transport, including the AB effect, and restricts the scope of future research on single TI nanowires. Therefore, a combined study using structural analysis, nano-ARPES^21^,^22^,^23^,^24^,^25^,^26^ and transport is proposed here in order to assess part of those remaining challenges on TI nanowires. Moreover, in contrast to a significant number of so far reported magnetotransport studies on Bi_2_Se_3_ or Bi_2_Te_3_ quasi-one-dimensional nanostructures, only few of them are devoted to Sb_2_Te_3_ nanowires. Hence, additional research contributions on Sb_2_Te_3_ based TI nanowires would enable a beneficial discussion on the nature of the observed magnetotransport features.

In this study, it is reported that the Φ_0_ periodicity occurs in quasi-one-dimensional Sb_2_Te_3_ nanowires grown by chemical vapor deposition. The combined study of magnetotransport experiments, together with nano-ARPES, indicates that the observed AB-type oscillations in the presence of a parallel magentic field (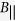) arise due to the presence of topologically protected two-dimensional surface channels and their contribution to the quantum transport effects in the Sb_2_Te_3_ TI nanowires. Moreover, nano-ARPES findings show the proximity of the Fermi level below the Dirac point, the intrinsic *p*-type doping of the nontrivial surface states as well as their Fermi velocity. The study includes magnetoresistance measurements of nanowires with different cross-sectional areas. The analysis of the magnetoconductance data with a magnetic field perpendicularly oriented to the wire axis (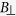) reveals universal conductance fluctuations (UCF) with characteristic changes of the correlation field *B*_*c*_ as the nanowire width is increased. The further extracted coherence length and temperature dependence of the UCF pattern provides information on the average limit of the coherent-transport within the wire and its quasi-one-dimensional character along the wire axis.

## Results and Discussion

Sb_2_Te_3_ nanowires (NW) are synthesized by Au-catalyzed chemical vapor deposition (CVD)^27^ in a quartz tube furnace (Lindberg/Blue M) with Sb and Te powder as source materials. The morphology and composition of as-grown Sb_2_Te_3_ nanowires are characterized by scanning electron microscopy (SEM) and energy dispersive X-ray spectroscopy (EDS, equipped in the SEM). The structural properties are studied by transmission electron microscopy (TEM) and powder X-ray diffractometry (XRD, CuKa, λ =1.5418 Å).

Figure 2(a) shows a typical SEM image of the as-grown Sb_2_Te_3_ nanowires. The corresponding EDS spectrum in Fig. 2(b) reveals that the atomic ratio of Sb and Te is about 2:3. Figure 2(c) shows the XRD spectrum of Sb_2_Te_3_ nanowires, verifying the rhombohedral structure of space group (R 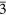
m) (JCPDS card No. 00-015-0874). Figure 2(d) shows a high-resolution TEM (HRTEM) image of a single Sb_2_Te_3_ nanowire taken along the [001] zone axis. Hexagonal fringes with inter-planar spacing of 0.21 nm for (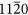) and (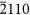) planes are labeled. The inset depicts the corresponding fast Fourier transform (FFT), confirming its single crystalline nature. The FFT is indexed and the growth direction is along (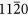), which corresponds to [110] in real space.

## Nano-ARPES Experiments

The micro- and nano-ARPES experiments with 120 *μ*m and 30 nm spot size, respectively have been carried out at the ANTARES beamline at the SOLEIL synchrotron, on a large set of single nanowire samples. Representative nano-ARPES measurements disclosing the band structure of a typical Sb_2_Te_3_ nanowire are shown in Fig. 3. Figure 3(a) shows the crystal lattice of the Sb_2_Te_3_ material exhibiting stacks of quintuple layers of Sb and Te along the main crystal axis. The corresponding reciprocal three-dimensional Brillouin zone with its respective surface projection indicates the 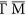 direction along which the nano-ARPES experiments in Fig. 3(b,c) were performed. The as-measured energy vs. momentum (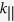) mapping along the 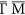 direction is shown in Fig. 3(b). Figure 3(c) shows the corresponding second derivative plot with respect to energy as a mean to observe better resolved band structure features. Similar to ARPES data on Sb_2_Te_3_ thin films, the band structure at binding energies around 0.5 eV, (accentuated by dashed white lines in Fig. 3) originates from bulk states. At the energy range between 0.2 eV and 0.4 eV the incoming surface band features might be mixed in. In fact, the two linear band dispersion crossing near the Γ-point are more clearly identified in the zoomed views of Fig. 3(d). They arise from the lower Dirac cone of the topologically protected surface states emerging from the nanowire surface. Since the Fermi energy (*E*_*F*_ = 0) cuts the Dirac cone slightly below the charge-neutrality point (Dirac point), [cf. Fig. 3(d)], only the lower part of the Dirac cone is revealed by nano-ARPES. From this high energy, angular and lateral resolution nano-ARPES data we have determined directly tiny, but important magnitudes of the electronic band structure of the investigated nanowires. In particular, the binding energy of the Dirac point located at 40 meV above the Fermi level, the hole doping level of about 2.5 × 10^12^ cm^−2^, and the Fermi velocity around 2.6 × 10^5^ m/s. Since *E*_*F*_ cuts the Dirac cone slightly below the Dirac point within the band gap of the bulk, *p*-type conduction with a significant contribution of the nontrivial surface states has to be considered in low temperature magnetotransport measurements.

For the transport measurements two ohmic contacts were fabricated on the Sb_2_Te_3_ NW according to the procedure described in the section for methods. Ti/Au electrodes formed ohmic contacts to Sb_2_Te_3_ nanowires with negligible contact resistance, serving as source and drain electrodes. Magnetoresistance measurements were performed on nanowires of different dimensions as listed in Table 1.

## Aharonov-Bohm Oscillations

Figure 4(a) shows magnetoresistance measurements between source and drain contacts of the nanowire W1 with the magnetic field applied parallel to the main-axis (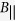). These are data after substraction of the parabolic background, using a zero-phase digital filtering and smoothing. Figure 4(a) (inset) reveals clear mirror symmetry of the spectra around zero magnetic field as well as periodic oscillations indicated by the dashed guidelines at the minima of the spectrum. A detailed view of the oscillations at positive magnetic field is displayed in Fig. 4(a,c) (main panel) for W1 and W2, respectively. The corresponding cross sectional area of the nanowires, measured by scanning electron microscopy, was found to be 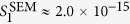 m^2^ for W1 and 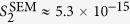 m^2^ for W2, [cf. insets of Fig. 4(b,d)], respectively. The oscillation period in W2 (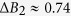 T) is found to be almost three times smaller than the period for W1 (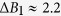 T). According to the theoretical description of AB-type oscillations, the total area encircled by the metal-like surface states and traversed by the magnetic flux, must be related to the oscillation period by 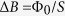
7,8. Here, *S* is the cross-sectional area of the wire. Therefore, the magnetic flux through the cross-section of the nanowires corresponds to 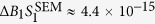 T m^2^ and 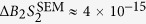 T m^2^, which results to be in good agreement with the theoretical value of the magnetic flux quantum 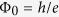. These results provide strong evidence that surface states arise around the TI Sb_2_Te_3_ NW, and effectively contribute to the conduction process along the nanowire channel. Possible bulk contribution to the quantum interference effects is here excluded due to the randomly distributed defect scattering centers in the bulk. These centers give rise to a large variety of different transport loops which indeed cause superposition and cancellation of the respective magnetoresistance oscillation patterns. Only those well defined loops around the NW surface survive, leading to a single well defined AB-type interference pattern. The Fourier transforms in Fig. 4(b,d) exhibit sharp frequency peaks at the corresponding frequencies of Φ_0_. Besides the Φ_0_ period, the minimum resistance due to weak antilocalization effects is observed at Φ = 0 for both samples. Note, that such a minimum, overlaps with the corresponding minimum of the equidistant series of the AB-type oscillations at zero magnetic field. Thus, although the AB-type minimum at zero field can not directly be observed in the spectrum, its reproducibility at zero field follows the respective periodicity. It corresponds to the expectation for TI surface state transport in intrinsically doped quasi-one-dimensional structures in the limit of weak or moderate strength of disorder^16^.

We rule out quantum transport through a surface hole accumulation layer as an origin for the AB-type oscillations: From the nanoARPES results in Fig. 3, we derive an intrinsic *p*-type doping of the Sb_2_Te_3_ nanowires. Since from experiment and theory, the Dirac point of the surface states energetically lies above the bulk valence band edge with an experimental accuracy of about 20–30 meV^28^, a small hole accumulation layer cannot be fully excluded. However, because of the quasi-metallic character of both, the *p*-doped bulk and a possible hole accumulation layer, electronic states in both regions are strongly coupled to each other so that confinement in the surface layer is suppressed.

In Fig. 4(a), weaker minima in between the maxima of the AB-type oscillations might be interpreted in terms of the Altshuler-Aronov-Spivak effect (AAS), which is theoretically characterized by the *h*/2*e* periodicity in magnetoresistance^29^. In one-dimensional TI systems this contribution is expected to be highly dependent on disorder strength. It is supposed to be almost absent in weakly disordered systems, while it becomes comparable or even starts to dominate over the *h*/*e* oscillation as the strength of disorder increases. Although in this experiment, a double frequency peak is not well resolved in the Fourier transform, a weak peak of the FFT at ≈0.9 T^−1^ in Fig. 4(b) could be attributed to a weak AAS contribution. The shift from the expected value might result from a non-exact alignment of the wire with respect to the applied parallel magnetic field during the measurement. The origin of these minima is still a matter of investigation. Measurements at different temperatures between 1.8 and 7 K for W2, (see Supplementary Fig. 1) unravel a systematic decrease of the oscillation amplitude, usually attributed to thermal broadening at higher temperature, due to phonon scattering.

## Universal Conductance Fluctuations

Below we will present and discuss the magnetoresistance measurements with the magnetic field applied perpendicular to the main axis of the nanowire (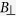). Figure 5(a) shows the corresponding magnetoconductance data for sample W3 at 1.8 K after the substraction of the parabolic background. The original magnetoresistance data are shown in Fig. 5(c). We see irregular magnetoconductance fluctuations up to high magnetic fields. These aperiodic variations in conductance are symmetric with respect to zero magnetic field [cf. Fig. 5(b)], and they are reproducible. The pattern is attributed to universal conductance fluctuations (UCF), rather than Shubnikov-de Haas (SdH) oscillations^30^,^31^, despite that ambiguous periodic-like features are observed in the 1/*B* dependence of the magentoconductance. We rule out SdH effects firstly, because for small nanowire widths, large magnetic fields would be required to produce cyclotron radii smaller than half the width of the wire (<100 nm in our samples). In fact, if the SdH effect would occur in the samples, they would not be observed at low magnetic field. Moreover, we also remark that for intrinsically doped samples, as it is expected in our nanowires due to intrinsic defects, impurity scattering reduces the mean free path so that the realization of SdH oscillations requires a high magnetic field to allow the carriers to complete a cyclotron orbit before any scattering event.

Following the UCF theory, relevant parameters of the nanowires have been extracted from the magnetoconductance autocorrelation function which is defined as: 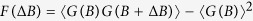 32. Here, 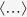 indicates an ensemble average. The correlation field *B*_*c*_ defined as the half-width at half-maximum of the correlation function: *F*(*B*_*c*_) ≡ *F*(0)/2, is a parameter describing the magnetic-field-induced dephasing of the interference phenomena. Figure 5(d) shows the conductance fluctuations of three representative nanowires, i.e., W3, W4 and W5 for 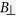 at 1.8 K. One finds that the correlation field *B*_*c*_, represented by the bars in Fig. 5(d), increases with decreasing nanowire width. Even more notable is the fact that as the width of the nanowire W3 is almost twice the value of the width of the nanowire W5, the corrrelation field of W3 (*B*_*c*_ ≈ 0.03 T), is reduced to about half the value of W5 (*B*_*c*_ = 0.057 T). Such an inverse proportionality ratio has also been observed for wire W4 (*B*_*c*_ = 0.039 T).

**Figure 1:**
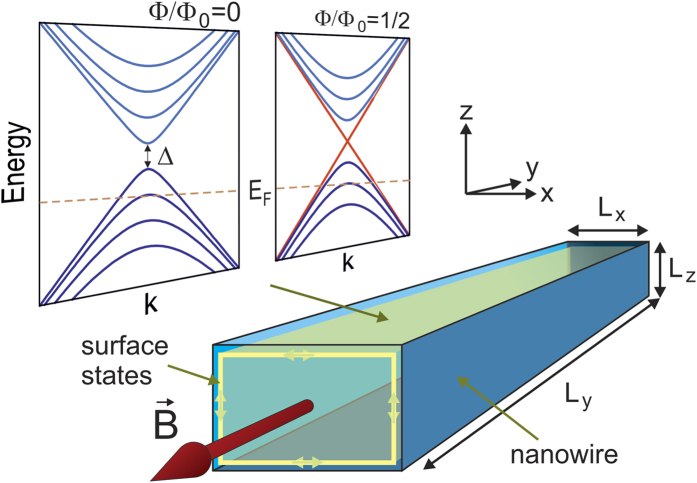
Schematic of the energy spectra of the nontrivial surface states in TI nanowires. On the bottom right the schematic of a TI nanowire describing metallic-like surface states around the cross-sectional area of the nanowire (yellow arrows), and traversed by the magnetic field parallel to *y*-axis. In the upper left the schematic of the surface state energy spectrum for 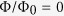 (gap opening) and 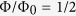 (closed gap).

**Figure 2:**
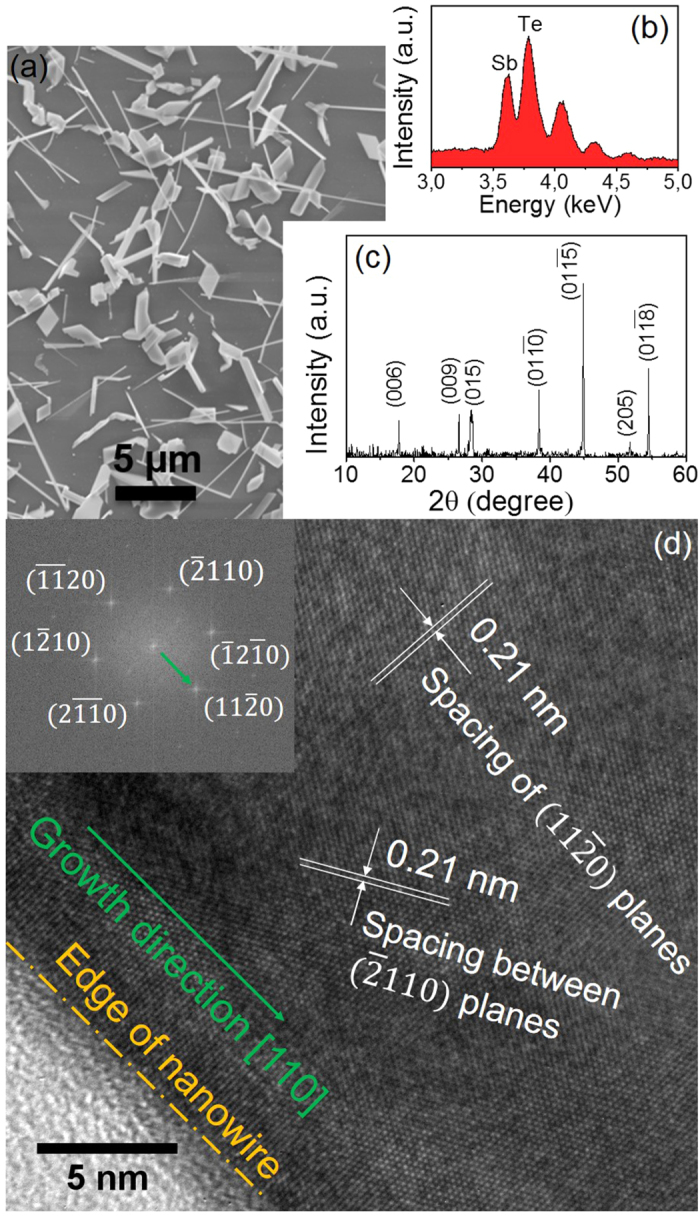
Material characterization of the Sb_2_Te_3_ nanowires. (**a**) SEM image of the as-grown Sb_2_Te_3_ nanowires. (**b**) The corresponding EDS spectrum. (**c**) XRD spectrum of Sb_2_Te_3_ nanowires. (**d**) HRTEM image of a single Sb_2_Te_3_ nanowire. Inset: the corresponding FFT.

**Figure 3:**
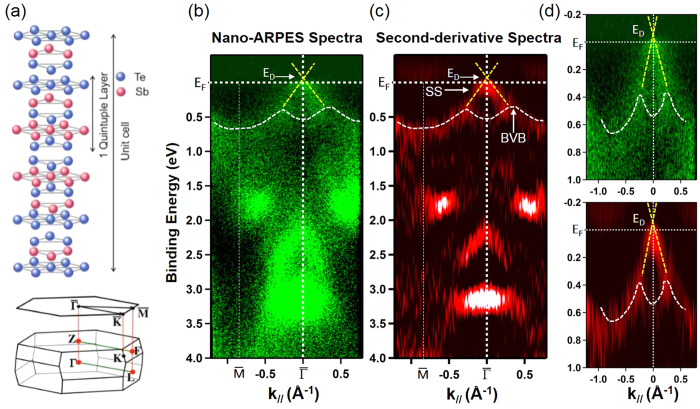
Selected nano-ARPES intensity maps along the 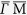 symmetry direction in the Brillouin zone of a typical Sb_2_Te_3_ nanowire. (**a**) Atomic structure and reciprocal three dimensional Brillouin zone with its respective surface projection for the Sb_2_Te_3_ material. (**b**) Energy vs. momentum (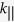) along the surface 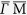 direction. (**c**) Second-derivative spectra of bands on panel (**b**). (**d**) Zoomed view around the Fermi level of spectra in panels b and c. The dashed yellow lines are a guide to the eye indicating the lower part of the Dirac cone (formed by topological surface states (SS)). Those SS are located above bulk valence bands (BVB) marked by dashed white lines.

**Figure 4:**
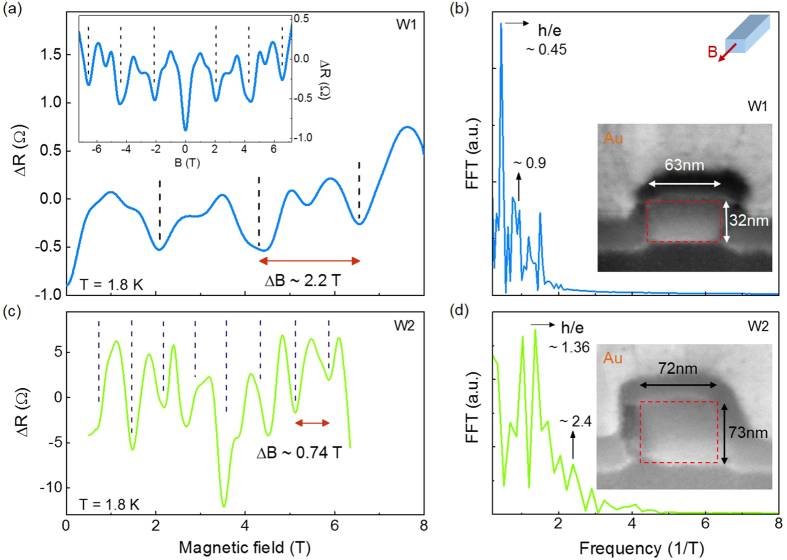
Aharonov-Bohm-type oscillations from the surface states in Sb_2_Te_3_ nanowires. (**a**) Magnetoresistance oscillations for nanowire W1 at 1.8 K. The magnetic field was oriented along the wire axis. The inset shows the periodic oscillations symmetric around zero. The dashed lines indicate the minima of the spectrum. (**b**) Corresponding Fourier transform and SEM image of the cross sectional area (inset). The period of 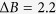 T fits to a single flux quantum (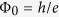) penetrating the nanowire cross section. (**c**) Magnetoresistance oscillations of the nanowire W2, with a larger cross section penetrated by a single flux quantum. (**d**) Corresponding Fourier transform and SEM image of the cross section (inset).

This correspondence yields a strong argument for the interpretation of the field-dependent magnetoconductance features in terms of UCFs. This is consistent with the theoretical prediction for *B*_*c*_ in one-dimensional systems in the dirty metal regime^33^,^34^:


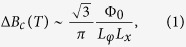


provided that the elastic mean free path *L*_*e*_ is smaller than all other relevant lengths (*L*_*e*_ < *L*_*z*_,*L*_*x*_ < *L*_*φ*_ < *L*_*y*_). *L*_*z*_ and *L*_*x*_ are the nanowire thickness and width, respectively, and *L*_*y*_ is the separation between the metallic contacts along the longitudinal axis of the nanowire [cf. Fig. 1]. Considering that *L*_*x*_ ≪ *L*_*y*_ is true for all the measured Sb_2_Te_3_ NW, it is then expected that the condition *L*_*φ*_ ≪ *L*_*y*_ is satisfied in the nanowires for the entire temperature range. Moreover, according to theory in one-dimensional systems the temperature dependence of *B*_*c*_(T) is entirely defined by *L*_*φ*_, independent of the relative lengths of *L*_*φ*_ and *L*_*T*_ (thermal diffusion length), as long as the sample dimensions fulfill the above mentioned limits. Thereby, in this study the coherence length *L*_*φ*_ has been extracted from equation (1). At 1.8 K *L*_*φ*_ was found to reach an average value of ≈900 nm with a maximum variation of about ±80 nm between different nanowire samples. With this estimation, *L*_*φ*_ appears to be larger than the measured perimeter around the rectangular cross-sectional area of the nanowires, which has been estimated to be close or smaller than 300 nm from SEM images [cf. Fig. 4(b,d) (inset)]. Then, even assuming both, surface states and bulk contribution to the UCF, the much larger average value of the coherence length points out that the transport due to metal-like surface states remains coherent throughout the loops encircling the cross-sectional area of the nanowire, when the magnetic field is applied parallel to the main axis (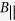). In this way, the crucial condition for the realization of the flux periodic oscillations, as shown in Fig. 4, is satisfied in our Sb_2_Te_3_ nanowires.

We first focus on the temperature dependence of the correlation field *B*_*c*_ and the coherence length *L*_*φ*_ for samples W3, W4, and W5 shown in Fig. 6. Keeping in mind the expression 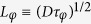, *B*_*c*_ arises solely from the changes in the phase relaxation time *τ*_*φ*_ and the diffusion constant *D*. The increase of the correlation field range *B*_*c*_ is consistent with a larger probability of inelastic scattering events at higher temperature and the respective decrease in coherence length. The phase relaxation mechanism of the surface states and intrinsic bulk carriers can be inferred from the temperature-dependent scaling of *L*_*φ*_. Here, typical sources of decoherence are assumed^35^. The electron-phonon contribution is expected to prevail at a temperature much higher than the observed experimental range (T < 6 K). Therefore, the phase-breaking time is mainly determined by electron-electron scattering events. Theory indeed, predicts that for low-dimensional disordered systems, especially in quasi-one-dimensional conductors, only electron-electron collisions involving small energy transfer represent the dominant relaxation mechanism (Nyquist mechanism). This mechanism is characterized by the length scale 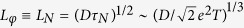 36, whose power law 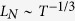 is consistent with the *T*^−0.36^ dependence of *L*_*φ*_ ≡ *L*_*N*_ shown in Fig. 6(b) for W5. Samples W3 and W4 exhibit a weaker dependence, *T*^−0.14^ and *T*^−0.11^ respectively, whose deviations from the expected theoretical dependence are similar to those found on quasi-one-dimensional, i.e. InN nanowires^37^.

Next we will discuss the evolution of the UCF amplitude upon increasing temperature. Figure 6(c) shows the conductance fluctuations in the sample W5 at different temperatures plotted in a low magnetic field range. The amplitude decreases as the temperature increases mainly due to the reduction of the phase-coherence length *L*_*φ*_, and the effect of thermal averaging at a length scale bigger than the thermal length 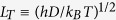, where 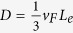 is the diffusion constant, *v*_*F*_ the Fermi velocity, and *L*_*e*_ the elastic mean free path. In spite of damping in amplitude, the “fingerprint” pattern is clearly reproduced up to ≈6 K. At 10 K the UCF features almost disappear. The magnitude of the conductance fluctuations is expressed by the root-mean-square of the conductance fluctuations amplitude rms (*G*). The temperature dependence of rms (*G*) is plotted in Fig. 6(d) for the three representative nanowires W3, W4, and W5. Since the separation between ohmic contacts (*L*_*y*_) is larger than the coherence lenght (*L*_*φ*_) in the measured samples, the estimated rms (*G*) includes contributions from different independent phase-coherent regions along the wire^38^. In this study we find that the resulting rms (*G*) values are consistent with some other theoretical and experimental magnetotransport results on quasi-one-dimensional TI structures at low temperature when *L*_*y*_ > *L*_*φ*_ 39,40,41.

**Figure 5:**
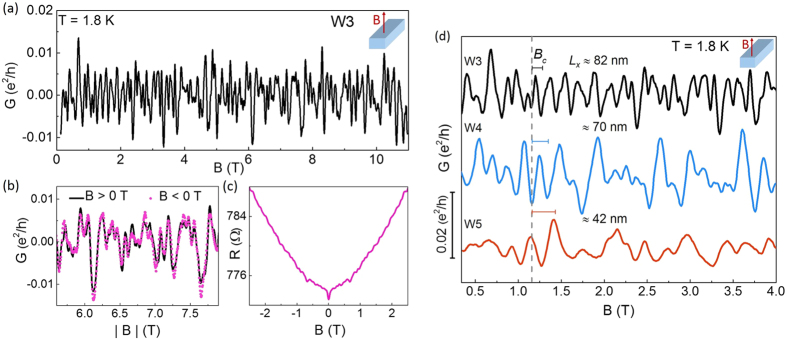
Universal conductance fluctuations at 1.8 K as a function of nanowire width. (**a**) Magnetoconductance fluctuations of nanowire W3 (in 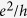 units) for a magnetic field applied perpendicular to the wire axis (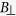). Data after substracting a parabolic background. (**b**) Superposition of the data for positive and negative magnetic field, solid black and dotted magenta lines, respectively. The data are shown only for a narrow field range for clarity. (**c**) Corresponding as-measured magnetoresistance data. (**d**) Comparison of magnetoconductance fluctuations for nanowires with different cross-sectional area: W3, W4 and W5 at 1.8 K for 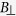. The spectra are offset for clarity. The dashed line and the respective bars, highlight the increase of the magnetic correlation field with decreasing nanowire width *L*_*x*_ (shown for each curve).

**Figure 6:**
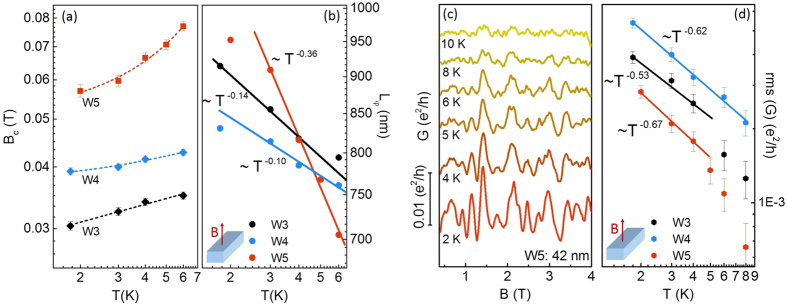
Temperature dependence of characteristic parameters determined from UCF. (**a**) Correlation field *B*_*c*_ vs. temperature for nanowires W3 (L_x_ ≈ 82 nm), W4 (L_x_ ≈ 70 nm), and W5 (L_x_ ≈ 42 nm). (**b**) Phase-coherence length 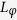 determined from *B*_*c*_ as a function of the temperature. The solid lines represent the fit to: 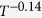 for W3 (black), 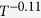 for W4 (blue), and 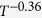 for W5 (red), respectively. (**c**) Magnetoconductance fluctuations of nanowire W5 at temperatures between 2 and 10 K. (**d**) Root mean square (rms) of the conductance fluctuations of nanowires W3, W4, and W5 as a function of temperature. The solid lines represent the power-law dependence (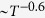) of rms(*G*) at low temperature.

Finally we analyse the temperature dependence of rms (*G*) for the three nanowires plotted in Fig. 5(d). Below ≈4 K, the data in Fig. 6(d) depict a power law dependence that is closely proportional to *T*^−0.6^ for the three samples. Unlike *B*_*c*_, which does not depend on thermal broadening, rms (*G*) depends on the relative value of *L*_*φ*_ and *L*_*T*_. Taking into account that *L*_*φ*_ < *L*_*y*_ in all measured samples, we consider the UCF theory for one-dimensional systems in two different regimenes: First, when *L*_*φ*_ < *L*_*T*_ < *L*_*y*_, the theory states: rms 
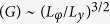
 42. Considering the temperature dependence of 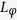 (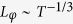), as it was extracted from 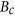, then, rms 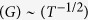. However, when *L*_*T*_ < *L*_*φ*_ < *L*_*y*_, rms (*G*) is expected to be largely affected by thermal broadening (energy averaging). In this case the theory predicts rms 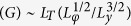
42. By substituting 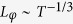 and 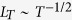 (from theory), we arrive at the temperature dependence rms 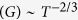 when 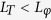 in one-dimensional systems. This last theoretical approach (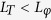) matches very well to the experimental temperature dependence of rms 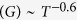 at the lowest temperature range, as it is presented in Fig. 6(d).

The present investigation has combined quantum transport experiments and the challenging nano-ARPES characterization, in order to provide further evidence on the existence of topologically protected surface states in intrinsically doped *p*-type Sb_2_Te_3_ nanowires. Although the Fermi energy still resides below the Dirac point due to the excess holes, the spectroscopy experiments have disclosed Dirac like states crossing the Fermi energy, which originate at the surface of the nanowire. The periodicity in magnetoresistance for 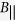, and its correlation with the geometric characteristics of the nanowires, indicate AB-type oscillations that can be consistently attributed to quantum transport effects within those topologically protected surface states around the nanowire. Therefore, despite bulk states might also contribute to the total conductance, nontrivial surface states in the Sb_2_Te_3_ nanowire would prevail in the realization of the observed AB-type oscillations. The origin of the magnetoconductance fluctuations at low temperature for perpendicularly oriented magnetic field (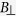) has been defined in terms of UCFs, as their characteristic parameters consistently change with the variations of the nanowire width according to the theoretical description for quasi-one-dimensional systems. The analysis of UCFs at low temperature confirms the coherent transport within a lenght scale larger than the AB-type surface paths.

## Methods

### Nanowire synthesis and device fabrication

The Si/SiO_2_ substrate coated with Au nanoparticles is placed downstream of the source materials in the furnace. The furnace is evacuated and flushed repeatedly with Ar gas. Then the furnace is heated to 430 °C for 6 hours. The Ar flow rate is kept at 80 sccm and the pressure is kept at ≈10 torr. The as-grown Sb_2_Te_3_ NW are suspended in isopropanol alcohol and drop-casted onto a Si/SiO_2_ substrate for electrical contact fabrication. Photolithography and electron-beam evaporation techniques are then used to define contacts (Ti/Au 20 nm/150 nm) to the single nanowires.

### Nano-ARPES measurements

The data have been obtained using linearly polarized photons with an energy of 100 eV. This “*k-microscope*” can be operated either on a point-mode manner or using the imaging-mode to create a two-dimensional image of the electronic states of interest. The Scienta acceptance plane is aligned along the ΓM symmetry direction with the acceptance angle set up either to 25° or 14° to obtain high angular resolution. The temperature during the photoemission measurements at ANTARES was kept at 100 K. The Sb_2_Te_3_ NW were transferred to a clean surface of a *p*-type silicon substrate. Ne ion sputtering (I = 38 mA, V = 1.5 kV) and subsequent annealing at 200 °C for 30 min was applied to prepare clean surfaces of the NW. The nanowires have been localized by using the mapping mode of the *k-microscope*. The chemical images are obtained by mapping the area of the core level peaks of Te 4*d* and Sb 4*d*.

### Magnetotransport measurements

Two-probe magnetoresistance was measured in a ^4^He cryogenic system using the standard low frequency AC technique with digital lock-in amplifiers. The magnetic field was varied from −13 T to 13 T. The AC voltage and bias current through the nanowire were kept below 200 *μ*V and 100 nA, respectively, in order to avoid electron heating and damage of the samples.

## Additional Information

**How to cite this article**: Arango, Y. C. *et al*. Quantum Transport and Nano Angle-resolved Photoemission Spectroscopy on the Topological Surface States of Single Sb_2_Te_3_ Nanowires. *Sci. Rep.* **6**, 29493; doi: 10.1038/srep29493 (2016).

## Supplementary Material

Supplementary Information

## Figures and Tables

**Table 1:** Dimensions of the Sb_2_Te_3_ nanowires. The column “separation” refers to the separation between the two Ti/Au contacts.

Nanowire	Width *L*_*x*_ (nm)	Thickness *L*_*z*_ (nm)	Separation *L*_*y*_ (*μ*m)
W1	63	32	1.8
W2	72	73	1.8
W3	82	54	1.8
W4	70	34	1.8
W5	42	29	1.8
