# Supplementary material for: Quantum Transport and Nano Angle-resolved Photoemission Spectroscopy on the Topological Surface States of Single Sb2Te3 Nanowires
Source: Sci Rep. 2016 Sep 1;6:29493. doi: 10.1038/srep29493 (PMC5007488; doi:10.1038/srep29493)
Supplement: Supplementary Information [file srep29493-s1.pdf]

Quantum Transport and Nano Angle-resolved Photoemission Spectroscopy on the Topological Surface States of Single Sb_2Te_3 Nanowires

Yulieth C. Arango[1*], Liubing Huang[2], Chaoyu Chen[3], Jose Avila[3], Maria C. Asensio[3], Detlev Grützmacher[1], Hans Lüth[1], J. Grace Lu[2,1] and Thomas Schäpers[1*]
[1]Peter Grünberg Institute (PGI-9) and JARA Jülich-Aachen Research Alliance, Research Centre Jülich GmbH, 52425 Jülich, Germany
[2]Department of Physics and Astronomy and Department of Electrophysics, University of Southern California, CA 90089, Los Angeles, USA and
[3]Synchrotron SOLEIL, L'Orme des Merisiers, Saint Aubin-BP 48, Gif sur Yvette 91192, France

Supplementary Figure 1: Magnetoresistance of the nanowire W2 at temperatures ranging from 1.8 to 7 K when the magnetic field is applied along the nanowire axis

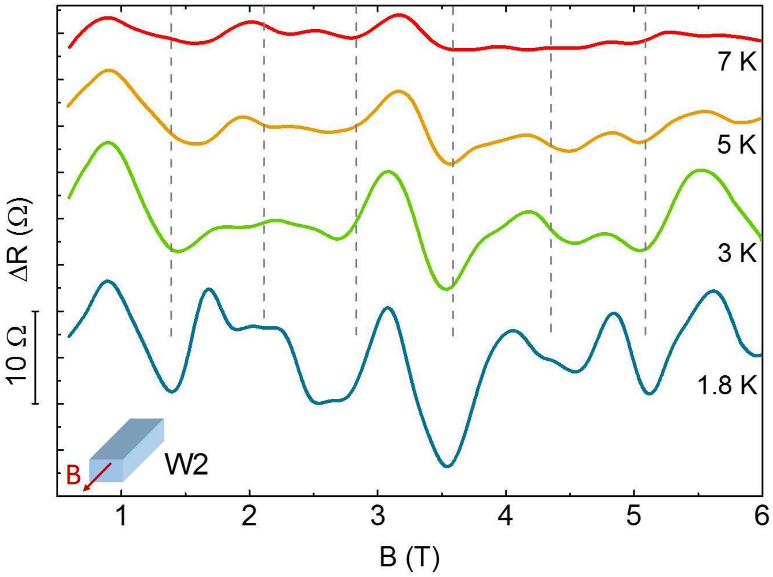

Figure 1: The position of the minima at the lowest measured temperature is indicated by the dashed lines. The data at higher temperature are incrementally shifted upward for clarity.

* Electronic address: y.arango@fz-juelich.de;th.schaepers@fz-juelich.de
